# Crocin ameliorates hepatic steatosis through activation of AMPK signaling in db/db mice

**DOI:** 10.1186/s12944-018-0955-6

**Published:** 2019-01-08

**Authors:** Li Luo, Kai Fang, Xiaomeng Dan, Ming Gu

**Affiliations:** 1 Department of Pharmacy, Union Hospital, Tongji Medical College, Huazhong University of Science and Technology, NO.1277 Jiefang Avenue, Wuhan, 430022 China; 2 Hubei Institute For Drug Control, Wuhan, 430075 China

**Keywords:** NAFLD, type 2 diabetes, crocin, AMPK, antioxidant

## Abstract

**Background:**

Non-alcoholic fatty liver disease (NAFLD) is closely linked to obesity, type 2 diabetes and other metabolic disorders worldwide. Crocin is a carotenoid compound possessing various pharmacological activities. In the present study, we aimed to investigate the effect on fatty liver under diabetic and obese condition and to examine the possible role of AMP-activated protein kinase (AMPK) signaling.

**Methods:**

db/db mice were administrated with crocin and injected with LV-shAMPK or its negative control lentivirus. Metabolic dysfunction, lipogenesis and fatty acid-oxidation in liver were evaluated.

**Results:**

In db/db mice, we found that oral administration of crocin significantly upregulated the phosphorylation of AMPK and downregulated the phosphorylation of mTOR in liver. Crocin reduced liver weight, serum levels of alanine aminotransferase, alanine aminotransferase, and liver triglyceride content, and attenuated morphological injury of liver in db/db mice. Crocin inhibited the mRNA expression of lipogenesis-associated genes, including sterol regulatory element binding protein-1c, peroxisome proliferator-activated receptor γ, fatty acid synthase, stearoyl-CoA desaturase 1, and diacylglycerol acyltransferase 1, and increased the mRNA expression of genes involved in the regulation of β-oxidation of fatty acids, including PPARα, acyl-CoA oxidase 1, carnitine palmitoyltransferase 1, and 3-hydroxy-3-methylglutaryl-CoA synthase 2. Moreover, treatment of crocin resulted in a amelioration of general metabolic disorder, as evidenced by decreased fasting blood glucose, reduced serum levels of insulin, triglyceride, total cholesterol, and non-esterified fatty acid, and improved glucose intolerance. Crocin-induced protective effects against fatty liver and metabolic disorder were significantly blocked by lentivirus-mediated downregulation of AMPK.

**Conclusions:**

The results suggest that crocin can inhibit lipogenesis and promote β-oxidation of fatty acids through activation of AMPK, leading to improvement of fatty liver and metabolic dysfunction. Therefore, crocin may be a potential promising option for the clinical treatment for NAFLD and associated metabolic diseases.

## Introduction

Non-alcoholic fatty liver disease (NAFLD) has become the most prevalent chronic liver disease affecting 15–40% of the general adult population [1]. NAFLD is closely linked to obesity, type 2 diabetes and other metabolic disorders worldwide [2], which is an important cause of cirrhosis and hepatic carcinoma worldwide [3]. In patients, NAFLD is defined as hepatic accumulation of lipid (mainly in the form of triacylglycerol) exceeds 5–10% of the total weight of liver [4]. Lipid accumulation in liver mainly depends on three sources, including intake of dietary fat, increased import of metabolites derived from lipolysis of fat in white adipose tissue, and fatty acids synthesized within the liver through de novo lipogenesis [5]. NAFLD includes a series of pathological changes ranging from hepatic steatosis, to abnormal hepatic lipid accumulation, and to cirrhosis, end-stage liver failure and hepatocellular carcinoma [6]. To date, no licensed drug or the treatment of NAFLD is available [7]. Therefore, it is important to develop new strategies for the intervention of NAFLD.

Although the accurate mechanism of NAFLD is not completely understood, increased generation of reactive oxygen species (ROS) is involved in the development of NAFLD [8]. Crocin, a carotenoid compound, is a bioactive constituent of both Saffron (*Crocus sativus* L.) and Gardenia plants [9]. A series of studies have verified the antioxidant activities of crocin [10–13]. Crocin has been reported to have a number of pharmacological activities, including anti-inflammatory, anti-cancer, neuroprotective, antihypertensive and cardioprotective effect [14]. There are evidence reporting that crocin can lower blood glucose level and insulin resistance, protect against obesity and ameliorate the lipid profile [15]. The protective effects of crocin against tissue damage in diabetes have been confirmed [13, 16–18]. Especially, Beshbishy et al. reported that crocin protects hepatic tissue against beryllium chloride-induced oxidative damage [19]. However, there has been little evidence on the effect of crocin on NAFLD-associated metabolic disorders.

In our preliminary studies, we found that crocin could upregulate the phosphorylation of adenosine 5′-monophosphate (AMP)-activated protein kinase (AMPK) and activate AMPK signaling. In this study we aimed to identify the beneficial effects of crocin on NAFLD and evaluated the hypothesis that crocin inhibit hepatic accumulation via activation of AMPK-dependent inhibition of lipogenesis and promotion of β-oxidation of fatty acids.

## Methods

### Animals experiments

All animal experiments were approved by the Institutional Animal Care and Use Committee of Union Hospital, Tongji Medical College, Huazhong University of Science and Technology (IACUCUH-2018-01-116) and in accordance with ARRIVE and NIH guidelines for animal welfare. C57BL/KsJ-Lepdb (db/db) mice and their lean littermates (wild type) were obtained from Mode Animal Research Center of Nanjing University. Eight-week-old male mice were maintained at 22 ± 2 °C with 60 ± 5% relative humidity, and under a 12 h light/dark cycle with free access to water and regular chow diet. db/db mice were randomly divided into three groups (db/db, Crocin, and Crocin+LV-shAMPK, *n* = 10 in each group). Mice in Crocin+LV-shAMPK group were injected with LV-shAMPK via tail vein at the age of 8 weeks. The injection of LV-shAMPK was performed once every two weeks for four weeks. Mice in db/db and Crocin group were injected with LV-shNC. After that, mice in Crocin and Crocin+LV-shAMPK groups were daily orally administrated with 20 mg/kg crocin for 8 weeks. Mice in db/db group was given vehicle administration. Meanwhile, WT mice was treated with vehicle in an identical manner as the normal control. After the treatment, all mice were anesthetized using 2% isoflurane and sacrificed. Blood samples were collected and tissue samples were snap-frozen in liquid nitrogen or collected for paraffin embedding.

### Histology examination of liver

Histological examination was performed to evaluated liver injury. Paraffin-embedded liver sections (5 μm) were prepared and stained with H&E. Images were captured using a light microscope (Olympus, Japan). Quantitative analysis of hepatic steatosis was performed using image-pro software and the percentage of vacuolar hepatocytes per field of vision was calculated. The results were shown as folds versus control.

### Metabolic and biochemical analysis

Before the OGTT and IPITT examinations, mice were fasted for 12 h and 6 h, respectively. Blood glucose level was measured at 0, 30, 60, 90, and 120 min after the administration of glucose and insulin. Blood glucose level was determined using tail vein blood via Accu-Chek (Roche, Basel, Switzerland). At the end of the experiment, mice were fasted for 12 h and blood samples were collected from the orbital venous plexus immediately before the sacrifice. Serum levels of alanine aminotransferase (ALT), alanine aminotransferase (AST), triglyceride (TG), non-esterified fatty acid (NEFA) and total cholesterol (TC), and liver TG were quantified using commercial kits (Nanjing Jiancheng, Nangjing, China). Serum insulin levels were determined using an enzyme-linked-immunosorbent assay (ELISA) kit (Invitrogen, Carlsbad, CA, USA).

### Real-time quantitative PCR

Total RNA from liver tissues was isolated using TRIzol (Life Technologies, Carlsbad, CA, USA). The RNA samples were treated with DNase and reverse-transcribed into cDNA using Superscript II (Life Technologies, Foster City, CA). Real-time RT-PCR was carried out with SYBR Green PCR Master Mix (Takara, Tokyo, Japan) in an ABI StepOnePlus Real-time PCR System. The expression of mRNA for each target gene was normalized relative to that of glyceraldehyde 3-phosphate dehydrogenase (GAPDH).

### Western blot analysis

Liver tissues were lysed in RIPA Lysis Buffer (Beyotime, Jiangsu, China). After the determination of protein concentration using BCA Protein Assay Kit (Beyotime, Jiangsu, China), the samples were separated with sodium dodecyl sulphate-polyacrylamide gel electrophoresis (SDS-PAGE) and transblotted onto polyvinylidene fluoride membranes (PVDF, Millipore). Subsequently, membranes were incubated with primary antibodies (diluted 1:1000; Cell Signaling Technology, USA) overnight at 4 °C. After washing with TBST, membranes were incubated with horseradish-peroxidase (HRP)-conjugated secondary antibody (diluted 1:5000; Thermo Fisher Scientific, USA) for 1 h at room temperature. Finally, the blots were visualized using chemiluminescence (ECL) detection reagents (Thermo Fisher Scientific, USA). Data were analyzed by calculation of integrated optical density.

### Statistical analysis

Results were expressed as the mean ± SD. The significance of differences among groups was assessed by one-way ANOVA analysis followed by Dunnett’s test. Statistical significance was defined as *p* < 0.05.

## Results

### Crocin upregulates AMPK signaling in liver of db/db mice

**Fig. 1:**
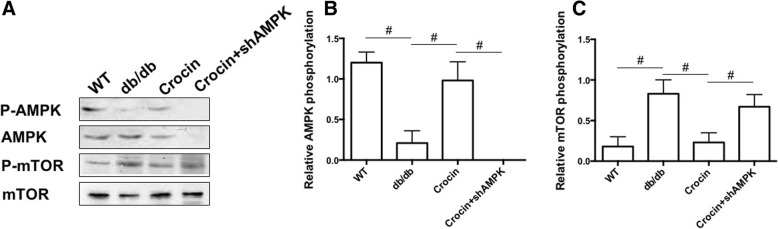
Effects of crocin on AMPK signaling in liver in db/db mice. **a** western blot analysis of AMPK and mTOR phosphorylation in liver tissue. **b** statistical analysis of AMPK phosphorylation (*n* = 3). (**c**) statistical analysis of mTOR phosphorylation (*n* = 3). The significance of differences among groups was assessed by one-way ANOVA analysis followed by Dunnett’s test. # *p* < 0.05, indicates statistical significance between the two groups

We firstly evaluated the effect of crocin on AMPK signaling in liver of db/db diabetic mice. As shown in Fig. 1a and b, AMPK phosphorylation was decreased in liver of db/db mice, compared with that in WT mice. Crocin markedly upregulated the phosphorylation of AMPK in liver of db/db mice (Fig. 1a and b). Crocin-induced enhancement of AMPK phosphorylation was significantly inhibited by LV-shAMPK (Fig. 1a and b). LV-shAMPK abolished the total protein expression of AMPK (Fig. 1a and b). Additionally, mTOR phosphorylation was increased in liver of db/db mice, compared with that in WT mice (Fig. 1a and c). Crocin resulted in a significant decrease of mTOR phosphorylation in liver of db/db mice, whreas this effect was suppressed by LV-shAMPK injection (Fig. 1a and c). These results demonstrated that Crocin upregulated AMPK signaling in liver of diabetic mice.

### Crocin attenuates lipid accumulation and injury of liver in db/db mice

**Fig. 2:**
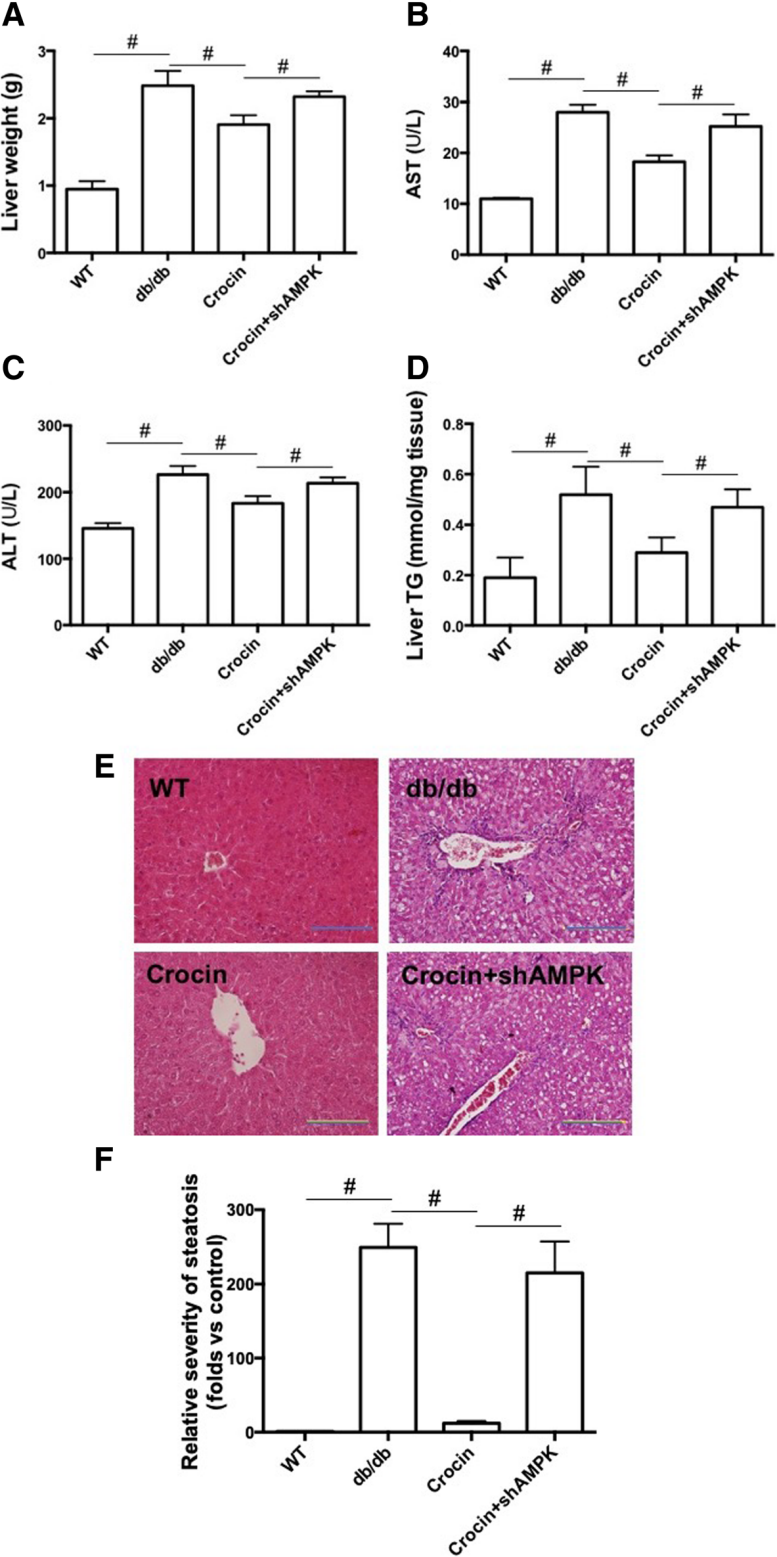
Effects of crocin on lipid accumulation and injury of liver in db/db mice. (**a**) liver weight (*n* = 10). (**b**) serum level of AST (*n* = 10). (**c**) serum level of ALT (*n* = 10). (**d**) liver content of TG (*n* = 10). (E) HE staining of liver tissue (*n* = 10). **f** Quantitative analysis was performed and expressed as folds of volume density (Vv) of steatosis versus control (*n* = 10). The significance of differences among groups was assessed by one-way ANOVA analysis followed by Dunnett’s test. # *p* < 0.05, indicates statistical significance between the two groups

In Fig. 2a, we showed that the increased liver weight in db/db mice was reduced by the treatment of crocin. Crocin-induced effect on liver weight was reversed by the injection of LV-shAMPK (Fig. 2a). The serum levels of AST and ALT in db/db mice were significantly higher than that in WT mice. Treatment of crocin remarkably reduced the serum levels of AST and ALT in db/db mice, which effect was inhibited by downregulation of AMPK (Fig. 2b and c). TG content was measured to evaluate lipid accumulation in liver. As shown in Fig. 2d, the high level of TG in liver of db/db mice was significantly reduced by crocin, whereas this effect of crocin was inhibited by downregulation of AMPK (Fig. 2d). Morphological changes of liver were observed and the results showed that there were marked vacuolar degradation and disorganized hepatocytes, and inflammatory infiltration in liver of db/db mice (Fig. 2e). Crocin significantly attenuated the morphological changes of liver and hepatic steatosis in db/db mice, which effect was inhibited by LV-shAMPK (Fig. 2e and f). These results suggested that crocin ameliorated hepatic steatosis and injury through activation of AMPK signaling in db/db mice.

### Crocin inhibits lipogenesis and promotes β-oxidation of fatty acids in liver in db/db mice

**Fig. 3:**
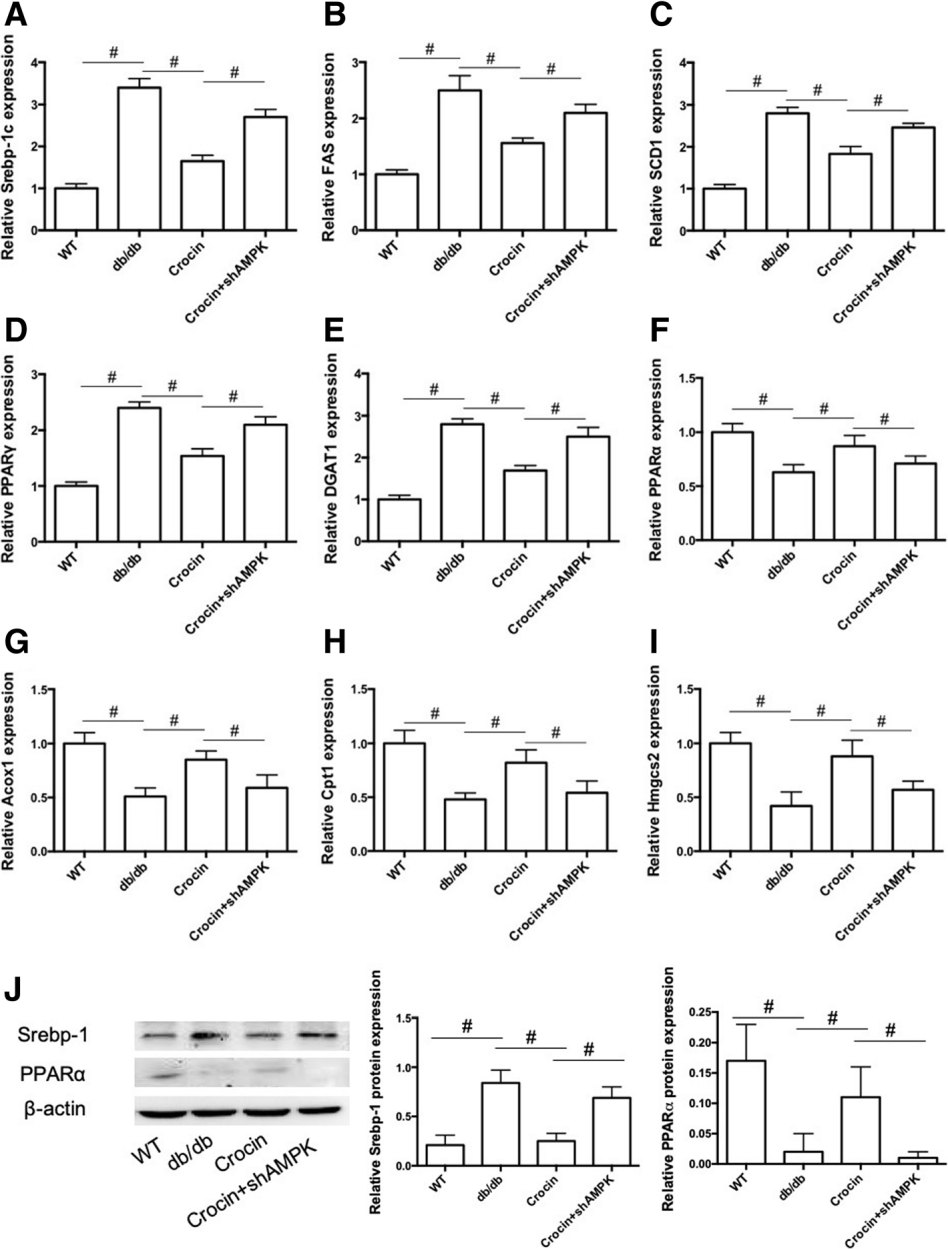
Effects of crocin on lipogenesis and β-oxidation of fatty acids in liver in db/db mice. mRNA expression of Srebp-1c (**a**), FAS (**b**), SCD1 (**c**), PPARγ (**d**), DGAT1 (**e**), PPARα (**f**), Acox1 (**g**), Cpt1 (**h**), and Hmgcs2 (**i**) in liver tissue were determined using real-time qPCR (*n* = 6). (J) Protein expression of Srebp-1 and PPARα was determined using western blot and statistical analysis was performed (*n* = 3). The significance of differences among groups was assessed by one-way ANOVA analysis followed by Dunnett’s test. # *p* < 0.05, indicates statistical significance between the two groups

To further evaluate the mechanism underlying the protective effects of crocin against hepatic steatosis and injury under diabetic condition, we determined the expression of a series of factors involved in the regulation of lipogenesis and β-oxidation of fatty acids. As shown in Fig. 3a-e, the mRNA expression of lipogenesis-related regulators, including sterol regulatory element binding protein-1c (SREBP-1c), fatty acid synthase (FAS), stearoyl-CoA desaturase 1 (SCD1), peroxisome proliferator-activated receptor γ (PPARγ), and diacylglycerol acyltransferase (DGAT) in liver of db/db mice, was significantly reduced by crocin. Crocin-induced decrease of mRNA expression of these factors was inhibited by LV-shAMPK (Fig. 3a-e). Moreover, the mRNA expression of β-oxidation of fatty acids-associated regulators, including PPARα, acyl-CoA oxidase 1 (Acox1), carnitine palmitoyltransferase 1 (Cpt1), and 3-hydroxy-3-methylglutaryl-CoA synthase 2 (Hmgcs2) in liver of db/db mice, was significantly increased by crocin (Fig. 3f-i). LV-shAMPK significantly inhibited crocin-induced upregulation of β-oxidation-related factors (Fig. 3f-i). In addition, crocin decreased SREBP-1 protein expression but increased PPARα protein expression in liver of db/db mice, which effect was inhibited by LV-shAMPK (Fig. 3j). These data demonstrated that crocin inhibited lipogenesis and promoted β-oxidation of fatty acids through activation of AMPK signaling in liver in db/db mice.

### Crocin mitigates systemic glucose and lipid metabolic disorder in db/db mice

**Fig. 4:**
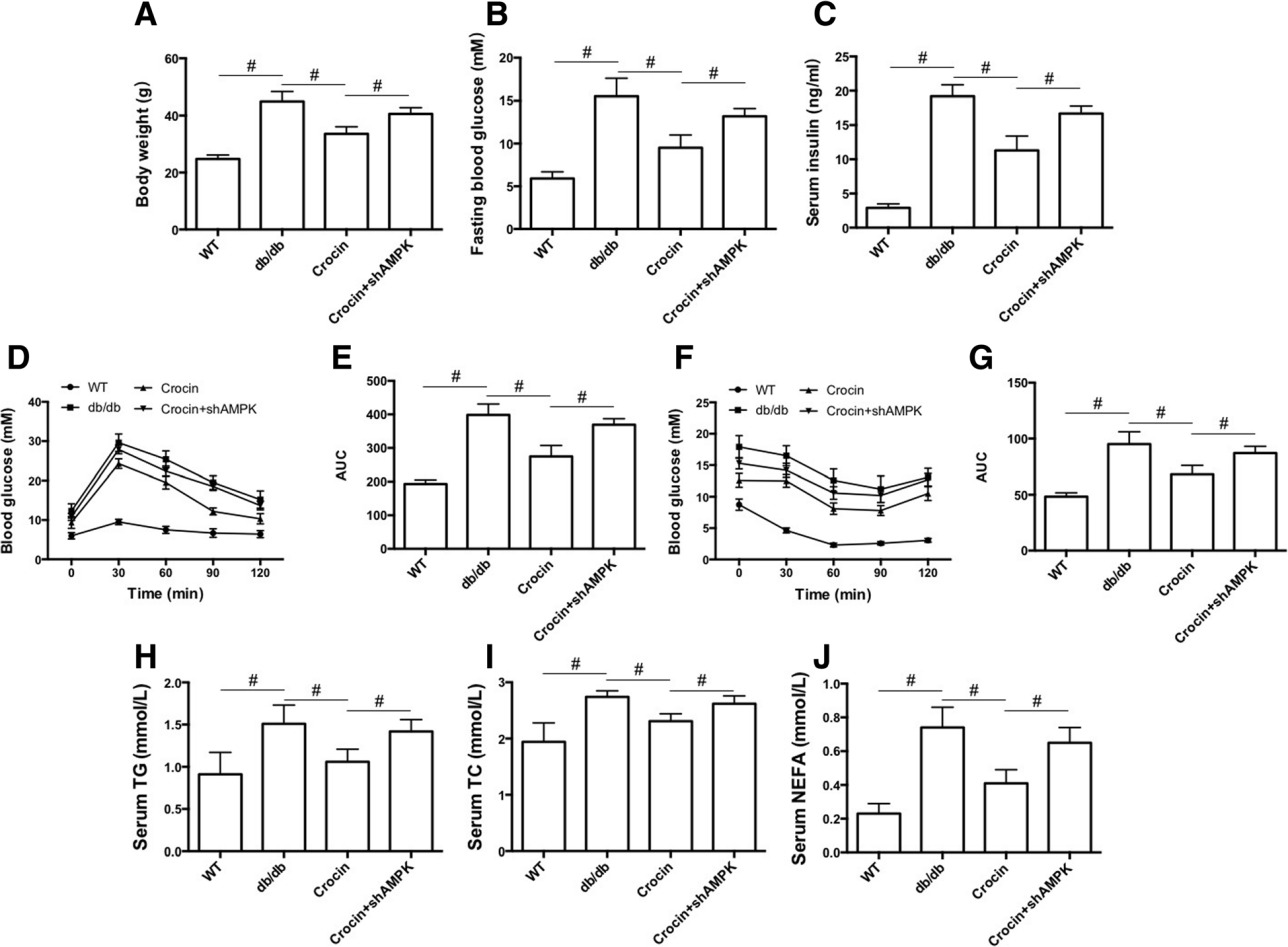
Effects of crocin on general glucose and lipid metabolism in db/db mice. (**a**) body weight (*n* = 10). (**b**) fasting blood glucose level (*n* = 10). (**c**) serum insulin level (*n* = 10). (**d**) OGTT test (*n* = 6). (**e**) area under the curve of OGTT (*n* = 6). (**f**) IPITT test (*n* = 6). (**g**) area under the curve of IPITT (*n* = 6). (**h**) serum level of TG (*n* = 10). (**i**) serum level of TC (*n* = 10). (**j**) serum level of NEFA (*n* = 10). The significance of differences among groups was assessed by one-way ANOVA analysis followed by Dunnett’s test. # *p* < 0.05, indicates statistical significance between the two groups

Finally, we evaluated the effect of crocin on general metabolism of glucose and lipid in db/db diabetic and obese mice. In Fig. 4a,b and c, we showed that crocin significantly decreased the high level of body weight, fasting blood glucose, and serum insulin, whereas LV-shAMPK notably suppressed these effects of crocin. We used OGTT and IPITT tests to evaluate the glucose tolerance and insulin sensitivity. As shown in Fig. 4d-g, in both OGTT and IPITT tests, crocin decreased the glucose level at different time points after the load of glucose and insulin and reduced the area under the curve (AUC). Crocin-induced attenuation of glucose metabolic disorder was inhibited by LV-shAMPK (Fig. 4d-g). Additionally, the levels of serum TG, TC and NEFA in db/db mice were significantly decreased by crocin, which effect was inhibited by LV-shAMPK. These results demonstrated that crocin attenuates glucose and lipid metabolic disorder through regulation of AMPK signaling in db/db mice.

## Discussion

NAFLD becomes a global epidemic and patients suffering from NAFLD display excessive accumulation of lipids in the liver, which is closely associated with non-alcoholic steatohepatitis (NASH), type 2 diabetes, cardiovascular diseases, and hepatocellular carcinoma [20, 21]. To date, there is no licensed drug for the treatment of NAFLD. Crocin is a natural constituent extracted from Saffron and Gardenia plants. Crocin has been verified to exhibit beneficial effects in diabetes. In the present study, we examined the possible protective effects of crocin and investigated the role of AMPK signaling. We found that crocin upregulated AMPK signaling and attenuated hepatic lipid accumulation and liver injury, as evidenced by decreased liver weight, serum levels of AST and ALT, liver TG level and morphological injury. Moreover, crocin ameliorated general glucose and lipid metabolic disorder.

NAFLD is believed to be the cumulative result of overactivation of lipogenesis and weakened fatty acid oxidation. de novo lipogenesis indicates synthesis of TG from 2-carbon precursors derived from glucose, fructose, and amino acids, which occupies over one-fourth of liver TGs [22]. A battery of key regulators are involved in the regulation of lipogenesis, including Srebp-1c, PPARγ, FAS, SCD1, and DGAT1 [23]. Knockdown of these key lipogenic enzymes has been shown to ameliorate hepatic steatosis-associated metabolic disorders, suggesting the essential role of inhibition of lipogenesis for the treatment of NAFLD [24, 25]. Srebp-1c is one of the most important transcriptional regulators controlling de novo lipogenesis, which function to regulate a series of lipid metabolism-related genes [26]. ob/ob mice with a hepatocyte-specific deletion of PPARγ are resistant to steatosis, indicating the essential role of PPARγ in the development of fatty liver [27]. FAS catalyzes the de novo synthesis of fatty acids and has long been categorized as a housekeeping protein, producing fat for storage of energy when nutrients are present in excess [28]. SCD1 catalyzes the synthesis of monounsaturated fatty acids, mainly oleate and palmitoleate, which are important in controlling weight gain in response to feeding high carbohydrate diets [29]. DGAT1 is proposed to have dual topology contributing to TG synthesis on both sides of the ER membrane and esterifying only the pre-formed fatty acids [30].

β-oxidation of fatty acids contribute to degradation of lipid in the liver and attenuation of TG accumulation, which process is regulated by pivotal regulators, such as PPARα, Acox1, Cpt1 and Hmgcs2. Dysfunction of hepatic lipid metabolism could be improved by activation of parenchymal cell PPARα through increasing ω-oxidation as well as peroxisomal and mitochondrial β-oxidation, which leads to enhanced hepatic transport, oxidation and metabolism of adipose tissue lipolysis-generated free fatty acids [31]. Cpt1 is required for the converting from fatty acyl-CoAs to their fatty acyl carnitine derivatives [32]. Acox1 participates in peroxisomal β-oxidation of fatty acid [33] and HMGCS2 regulates mitochondrial fatty acid oxidation [34].

AMPK is an important regulator of fat metabolism in the liver. Downregulation of AMPK has been found to increase lipogenesis and reduce fatty acid oxidation [21]. Overexpression of AMPK in liver decreases gene expression involved in lipogenesis, TG content in liver, and hepatic steatosis, while increasing fatty acid oxidation-associated gene expression and promoting β-oxidation of fatty acids [35, 36]. Among those lipid metabolism-related regulators, AMPK has been reported to interact with PPARs in the context of fatty liver. For instance, palmitoleic acid improves metabolic functions in fatty liver by PPARα-dependent AMPK activation [37]. Adiponectin increases fatty acid oxidation in skeletal muscle cells by sequential activation of AMPK/p38 PPARα [38]. In our study, we found that activation of AMPK by crocin could inhibit PPARγ expression but increase PPARα expression. Considering the important role of PPARs in the regulation of lipid metabolism, the interaction between AMPK and PPARs may be crucial for the beneficial effect of crocin.

Hepatic lipid accumulation is closely associated with the development of insulin resistance and glucose metabolic dysfunction [39]. Thus, crocin-induced amelioration of fatty liver may contribute to improvement of glucose metabolic dysfunction. Moreover, crocin-resulted in a reduction of body weight, indicating that crocin may influence the formation or accumulation of adipose. Further studies are needed to evaluate the effect of crocin on obesity and adipose tissue.

In summary, in our study, we showed that inhibition of AMPK significantly inhibited the protective effects of crocin against hepatic steatosis and metabolic dysfunction. Downregulation of AMPK significantly suppressed crocin-induced downregulation of lipogenesis-related regulators and upregulation of fatty acid oxidation-associated regulators. The results suggest that AMPK signaling plays a crucial role in the protective effects of crocin against NAFLD, which may be attributed to the regulation of lipogenesis and β-oxidation of fatty acids.

## Conclusions

The data from this study suggest that crocin could alleviate and modulate obesity-associated NAFLD in db/db mice. Crocin can inhibit lipogenesis and promote β-oxidation of fatty acids through activation of AMPK. Therefore, crocin may be a potential promising option for the clinical treatment for NAFLD.

## Abbreviations

Acox1: acyl-CoA oxidase 1
ALT: alanine aminotransferase
AMPK: adenosine 5‘-monophosphate (AMP)-activated protein kinase
AST: alanine aminotransferase
AUC: area under the curve
Cpt1: carnitine palmitoyltransferase 1
DGAT: diacylglycerol acyltransferase
FAS: fatty acid synthase
Hmgcs2: 3-hydroxy-3-methylglutaryl-CoA synthase 2
NAFLD: non-alcoholic fatty liver disease
NEFA: non-esterified fatty acid
PPARγ: peroxisome proliferator-activated receptor γ
ROS: reactive oxygen species
SCD1: stearoyl-CoA desaturase 1
SREBP-1c: sterol regulatory element binding protein-1c
TC: total cholesterol
TG: triglyceride
